# Photosensitive Poly-l-lysine/Heparin Interpolyelectrolyte Complexes for Delivery of Genetic Drugs

**DOI:** 10.3390/polym12051077

**Published:** 2020-05-08

**Authors:** Viktor Korzhikov-Vlakh, Iuliia Katernuk, Iuliia Pilipenko, Antonina Lavrentieva, Ivan Guryanov, Vladimir Sharoyko, Alina A. Manshina, Tatiana B. Tennikova

**Affiliations:** 1 Institute of Chemistry, Saint Petersburg State University, St. Petersburg, Universitetskii pr. 26, Peterhoff 198504, Russia; katernuk02@gmail.com (I.K.); yulia-sobinina@ya.ru (I.P.); ivan.guryanov1@gmail.com (I.G.); sharoyko@gmail.com (V.S.); a.manshina@spbu.ru (A.A.M.); tennikova@mail.ru (T.B.T.); 2 Institute of Technical Chemistry, Leibniz University, Callinstrasse 5, 30167 Hannover, Germany; lavrentieva@iftc.uni-hannover.de

**Keywords:** poly-l-lysine, heparin, interpolyelectrolyte complex, photosensitive, siRNA, pDNA

## Abstract

Photo-triggered release of biopharmaceutical drugs inside the cells is a challenging direction of modern science, which requires obtaining new polymeric systems. The interpolyelectrolyte complexes (IPECs) of poly-l-lysine with heparin capable of encapsulation of genetic constructions—such as model oligonucleotide, siRNA, and pDNA—were obtained. Poly-l-lysine to heparin ratios were optimized to provide the appropriate release kinetics of genetic material from the polyplex. In order to impart the obtained IPEC with photosensitive properties, the linker was synthesized as based on 4-brommethyl-3-nitrobenzoic acid. The conditions and kinetics of photosensitive linker destruction were carefully studied. The colloid particles of IPEC were modified with Cy3 probe and their cellular internalization was investigated by flow cytometry method. The efficacy of photosensitive IPECs as siRNA and pDNA delivery system was evaluated.

## 1. Introduction

Gene therapy represents the actual and emerging area of interdisciplinary research, which aims at treatment of wide range of pathologies, from monogenic genetic disorders to inherited and acquired human diseases [1]. The main idea of such medical treatment is to deliver missing genes to the cells in order to initiate the synthesis of missing proteins to normalize biochemical processes in the organism. The fast development of this area started more than 30 years ago with application of viral vectors, but some negative clinical cases [2,3,4] caused some disappointment. However, during last decade, interest in gene therapy is growing again, involving more and more research [5,6,7]. Different types of nucleic acid therapies have been developed: gene sequences for initiation of missing protein synthesis (plasmid DNAs—pDNAs [8] and minicircle DNAs—mcDNAs [9,10]) and those for inhibition of protein translation (small interfering RNAs—siRNAs [11], aptamers [12] and antisense oligonucleotides [13]). The challenging task is to provide the entry of the effective genetic construction inside the target cells in vivo as well as to ensure its safe release in order to affect the genetic apparatus of the cells. Adeno-associated viral (AAV) vectors have great promise but they experience certain limitations and require special conditions to be used in clinics [1,14]. Also, the application of viral vectors is known to be potentially immunogenic [15] and expensive [16]. 

The above-mentioned factors motivate to the development of non-viral vectors, which could be potentially used for in vivo treatment of patients. There are a number of non-viral systems for delivery of genes into the cell: liposomes [17], niosomes [18], cationic lipids (lipoplexes) [19], cationic polymers (polyplexes) [20,21], and polypeptides [22]. Among these systems, polycations attract the significant interest due to their ability to form interpolyelectrolyte complexes (IPECs) with DNA or RNA. Such polyplexes could be characterized by high storage stability and quite narrow polydispersity [23]. Moreover, they provide the compaction of DNA, similarly to that in chromosome, which result in protection of genetic construction from degradation and possibility of internalization into the cells [24]. There are a number of polycations, which showed interesting properties as gene delivery vehicles [20], among which poly(ethyleneimine) [25,26], poly-l-lysine (PLL) [22,27,28] and chitosan [29,30] are the most studied ones. The high charge density of polycations, improved the binding of DNA and cell penetration, but could not provide the effective intracellular unpacking of genetic construction to release in cytoplasm and affect the inner biochemistry of cells [21,30,31]. In order to diminish this effect, different polyanions were considered as a competitive counter-ions [29,32,33]. In our recent research, we have enhanced the transfection of pEGFP and anti-VEGF siRNA with chitosan by addition of heparin as a competitive polyanion [29]. Other approaches include the application of polycations covalently modified by polyanions to enhance gene delivery efficacy [26,34]. The combination of positively charged (lysine) with negatively charged (glutamic acid) and hydrophobic amino acids in one polypeptide chain seemed also to be interesting for the realization of genetic drugs’ delivery approach [22]. 

In recent decades, the idea of photo-triggered release of genetic constructions inside the target cells was examined [25,35,36]. The inspiration of such approaches is to increase the control over the cytosolic release of genetic therapeutics. This could be achieved by photo-sensitivity of externally added components, such as indocyanine green, disturbing the structure of liposomes upon irradiation with electromagnetic radiation with certain wavelength [35], or by utilization of photosensitive compound as linker between two blocks of block-copolymer, which is decomposed upon exposure to such radiation [37]. Both disturbance of liposomes and decomposition of block-copolymers result in rapid release of DNA or RNA into the cytosol. The triggered release of siRNAs by photosensitive destruction of polymer chain of polycations within polyplexes was also described [38,39]. However, to the best of our knowledge, the possibility of triggered release of the genetic drugs from interpolyelectrolyte complexes cross-linked by photosensitive linker was not yet studied. 

As it was already mentioned above, the strong polyanion—such as heparin sulfate—can displace DNA from the polyplex by competing interactions with strong polycation [29,40,41]. Based on this observation, we have prepared the nanogels based on PLL complexes with heparin (Hep), which were capable of encapsulation and release of pDNA and siRNA. The composition of IPECs was optimized and the strategy for cross-linking of such particulate formulations with preliminary synthesized photosensitive linker based on 4-brommethyl-3-nitrobenzoic acid was developed. The photosensitive release of genetic constructions from cross-linked IPECs obtained was studied. Additionally, their ability to penetrate inside the cells and deliver model siRNA and pDNA was evaluated.

## 2. Materials and Methods 

### 2.1. Materials

*ε*-*Z*-l-Lysine (>99%), triphosgen (99%), *α*-pinene (99%), *n*-hexylamine (99%), trifluoroacetic and trifluoromethanesulfonic acids (99.9%), 4-bromomethyl-3-nitrobenzoic acid (98%), *N*-(3-dimethylaminopropyl)-*N*′-ethylcarbodiimide hydrochloride (98%, EDC), fluorescein isothiocyanate (FITC), *N*-hydroxysuccinimide (98%, NHS), Schiff’s reagent and heparin sodium salt (*M_w_* 8000–12,000) were ordered in Sigma-Aldrich (Darmstadt, Germany). *N*-Boc-1,2-diaminoethane (98%) was the product of Acros Organics (Geel, Belgium). NHS-esters of Cy3 and Cy5 probes were purchased in Lumiprobe (Moscow, Russia). Ethidium bromide (EtBr) was purchased from Molecular Probes (Eugene, OR, USA). These and other common chemicals, which were utilized in this work were used without special purification. 

SEC column calibration was performed with the use of poly(methyl methacrylate) (PMMA) standards (*M_w_* 17,000–250,000; *Đ* ≤ 1.14) purchased from Supelco (Bellefonte, PA, USA). Spin columns (molecular weight cut-off (MWCO) 3000; VivaScience, Sartorius Group, Göttingen, Germany) and Eppendorf tubes with filter (30,000 MWCO, Amicon Ultra 0.5 mL, Merck, Darmstadt, Germany) were used for dialysis, IPECs purification, and separation.

Cy3-labeled 23-base pairs duplex of oligothymidine and oligoadenine (oligo-dT-dA) was purchased from Biobeagle (St. Petersburg, Russia). siRNA directed against GFP messenger RNA (sense strand: 5′-GCAAGCUGACCCUGAAGUUCAU-3′, antisense strand: 5′-GAAC UUCAGGGUCAGCUUGCCG-3′) was obtained from IBA (Göttingen, Germany). Plasmid encoding luciferase pCLuc4 was a kind gift of Dr. Marika Ruponen (School of Pharmacy, University of Eastern Finland, Kuopio, Finland). Branched poly(ethylene imine) (PEI; *M_w_* = 25,000 and *M_n_* = 10,000 according to GPC; Sigma-Aldrich, St Louis, MO, USA) was used as control for gene silencing and transfection studies.

Cells were cultivated in Dulbecco’s modified Eagle medium (DMEM) supplemented with 10% (*v/v*) fetal calf serum (FCS), and 1% (*v/v*) penicillin/streptomycin (P/S) in humidified environment at 37 °C, 5% CO_2_. All medium components were purchased in Biochrom GmbH (Berlin, Germany). The plastic for cell cultivation was obtained from Sigma-Aldrich (Darmstadt, Germany) and Sarstedt AG&Co (Nümbrecht, Germany).

CellTiter-Blue dye (CTB) was product of Promega (Madison, WI, USA). SlowFade™ Gold Antifade Mountant with DAPI dye and Gaussia Luciferase kit were obtained from Thermo Fisher Scientific (Waltham, MA, USA). 

### 2.2. Instruments

The magnetic stirrer MR Hei-Mix S (Heidolph, Schwabach, Germany), Schlenk reaction tubes with rubber septum (Aldrich, Munich, Germany) and rotary evaporator Hei-VAP Precision ML/G3B (Schwabach, Heidolph, Germany) were used for polymer synthesis. NMR spectroscopic data were recorded with equipment of Magnetic Resonance Research Centre of St. Petersburg State University: Bruker Avance spectrometer (400.13 MHz for 1H and 100.61 MHz for 13C) in DMSO-d6 or in CDCl_3_ and were referenced to residual solvent proton signals (δH = 2.50 and 7.26 ppm, respectively). Shimadzu LC-20 Prominence system supplied with refractometric RID 10-A detector (Kyoto, Japan) and 7.5 × 300 mm Agilent PLgel MIXED-D column (Chrom Tech, Apple Valley, MN, USA) were applied for SEC analysis. 

An ultrasound homogenizer (Sonopuls HD2070, Bandelin, Berlin, Germany) and TS-100C thermo-shaker BioSan (Riga, Latvia) were applied for interpolyelectrolyte complexes formation and particles’ modification reactions. The Vivaspin column ultra-filtration and particle separation were performed with Sigma 2–16 KL centrifuge (Sigma, Darmstadt, Germany).

A dynamic light-scattering (DLS) instrument Zetasizer Nano ZS, Malvern, Enigma Business Park (UK) was used for measurements of surface charge, particle size, and particle size distribution. siRNA and pDNA concentrations were quantified spectrophotometrically with application of Nanodrop 2000c spectrophotometer (Thermo Fischer Scientific, Vantaa, Finland). The photometrical detection of aldehyde groups was performed with UV–Vis-1800 Shimadzu (Kyoto, Japan). Microplate Spectrophotometer-Fluorometer Fluoroskan Ascent reader (Thermo Fisher Scientific, Waltham, MA, USA) was used for quantitative analysis of fluorescence during linker decomposition studies. 

IPECs morphology was analyzed in Centre for Molecular and Cell Technologies of St. Petersburg State University by using transmission electron microscope Zeiss Libra 200FE (Carl Zeiss, Oberkochen, Germany). 

Agarose gel electrophoresis was performed with BlueMarine 200 system (Serva Electrophoresis GmbH, Heidelberg, Germany) and analyzed with SkyLight Table ECX-F20 (Vilber, Collégien, France). The experiments on Microscale Thermophoresis were carried out with a Monolith NT.115 (Nanotemper, Munich, Germany). The Monolith NT.115 Premium capillaries were used.

The HCCL-30UM(I) and HCCL-8UM(I) He-Cd laser systems (Plasma JSC, Ryazan, Russia) generating laser with 325 nm wavelength with intensities 3 W/cm^2^ and 0.8 W/cm^2^, respectively, were used for the photo triggered linker decomposition studies. Also, the UV LED lamp constructed by Jaakko Itkonen (Pharmaceutics and Biopharmaceutics Department, University of Helsinki, Finland) as based on 5W UV 365 nm High Power LED DC 7 V–7.5 V, 700 mA TopLedLight lamp (Shenzhen, China) was used for linker decomposition studies.

Cell morphology and cellular internalization of IPECs were analyzed with Olympus IX50 fluorescent microscope (Olympus Corp., Tokyo, Japan). Flow cytometry was performed with application of BD Accuri C6 (Becton Dickinson, Franklin Lakes, NJ, USA) supplied with a 488 nm argon-ion laser. Red light of particles fluorescence was collected by a 670 long-pass filter. The data were analyzed with BD Accuri C6 Software (v. 1.0, Becton Dickinson, Franklin Lakes, NJ, USA, 2011).

### 2.3. Cells 

HEK-293 (human embryonic kidney), BEAS-2B (human bronchial epithelium), and NIH 3T3 (expressing GFP mouse fibroblast cells) cell lines were obtained from the German Collection of Microorganisms and Cell Culture (DSMZ, Braunschweig, Germany). HCE-T (human corneal epithelial cells) were a kind gift of Prof. Arto Urtti (School of Pharmacy, University of Eastern Finland, Kuopio, Finland). 

### 2.4. Methods

#### 2.4.1. Poly-l-lysine Synthesis

The synthesis of poly-l-lysine with different molecular weights was performed via *n*-hexylamine initiated ring-opening polymerization of corresponding NCA in anhydrous 1,4-dioxane as described elsewhere [22]. The reaction was carried out at 55 °C during 4 h. The product was precipitated with an excess of diethyl ether, then the precipitate was filtrated, washed with diethyl ether, and then dried. The characteristics of the products are presented in Table S1.

#### 2.4.2. Photosensitive Linker Synthesis

To a solution of 4-bromomethyl-3-nitrobenzoic acid (260 mg, 1 mmol) in 10 mL of acetonitrile *N*-Boc-1,2-diaminoethane (0.2 mL, 1.3 mmol) was added. The reaction mixture was intensively stirred during 5 h at 65–70 °C in a flask equipped with a reflux condenser. The reaction product appears in the form of precipitate. The precipitate was isolated by filtration using a Schott filter #16, washed with acetonitrile, and dried in air for 2 h. The yield was 282 mg (83%). ^1^H NMR (400 MHz, DMSO-d6): δ 8.28 (s, 2H); 8.15 (s, 1H); 7.78 (d, 2H); 4.04 (s, 2H); 3.52 (m, 2H); 2.59 (m, 2H); 1.35 (s, 9H).

#### 2.4.3. IPECs Preparation

Non-cross-linked IPECs preparation: IPECs were prepared using the method of polyelectrolyte complexation similarly to described in [29]. Poly-l-lysine (PLL) and heparin (Hep) 1 mg/mL solutions in 0.1 M phosphate buffered solution, pH 7.4, were prepared. Then the solutions of polycation and polyanion were diluted in order to get 1 mL of solutions with concentrations, which will enable achieving the desirable molar ratio of charged groups in the system upon mixing. Before the complexation, PLL solution was sonicated during 30 s and 10% power. Then the solution of Hep was then dropwise added by syringe under sonication at 20% power. The obtained colloid system was stirred at 350 rpm using a ThermoShaker for 30 min at room temperature. 

Encapsulation of genetic cargo: to encapsulate oligo- and polynucleotides inside PLL-Hep IPECs, the anionic cargo was added to PLL solution immediately after sonication under stirring, thereafter heparin was added to these polyplexes. In order to separate the obtained IPECs from free oligo- and polynucleotides the suspension was centrifuged at 15,000 *g* during 7 min at 4 °C and redispersed in distilled water. The procedure was repeated 3 times. In the case of Cy3-oligo-dT-dA the fluorescence of the supernatant (λ_ex_ = 550 nm, λ_em_ = 570 nm) was measured and encapsulation efficacy (*EE*, %) was estimated as follows:

*EE* = ((*q_initial_* − *q_supernatant_*)/*q_initial_*) × 100% (1)

where *q_initial_* is initial quantity of oligonucleotide taken for complexes preparation and *q_supernatant_* is the total quantity of the oligonucleotide detected in supernatant solution with application of previously plotted calibration curve (Figure S1). The EE of plasmid DNA was evaluated in a similar way. The concentration of polynucleotide was quantified by the measurement of UV absorbance at 260 nm. 

Cross-linked IPECs preparation: In order to obtain IPECs cross-linked by photosensitive linker the PLL-linker conjugate was firstly obtained. For that the activated esters method of linker carboxylic group activation was applied. Briefly, 4 mg of 4-(((2-((tert-butoxycarbonyl)amino)ethyl)amino)methyl)-3-nitrobenzoic acid (linker) was dissolved in 200 μL DMSO and the quantity needed was taken into the reaction (see the [PLL]:[Hep]:[linker] molar ratios in Results and Discussion). 500 μL of NHS (2.71 mg, 0.0236 mol) in 0.01 M MES buffered solution, pH 5.5, was added to the solution of the linker, and after 5 min, 500 μL of EDC (3.66 mg, 0.0236 mol) in the same buffered solution was added. The activation reaction was carried out at 0 °C during 60 min with constant stirring (500 rpm). After that, 1 mL of PLL solution (1 mg/mL) in 0.1 M borate buffered solution, pH 10 was added to the mixture. The solution was left under 350 rpm stirring at room temperature overnight, after which the unbound linker was removed by ultra-filtration (MWCO 3000), followed by washing with water. The total quantity of unbound linker in the supernatant (*q_supernatant_*) was determined spectrophotometrically with application of calibration curve (Figure S2) and the quantity of bound linker (*q_bound linker_*, %) was calculated similar to Equation (1)

*q_bound linker_* = ((*q_initial_* − *q_supernatant_*)/*q_initial_*) × 100% (2)

Then the Boc protection was removed by trifluoroacetic acid treatment as described elsewhere [42] and the complex with oligo- or polynucleotide was formed according to the procedure, which was related above. The Hep with EDC/NHS activated carboxylic groups was applied on this stage. The activation of Hep was performed similarly to the procedure described above for the linker activation. EDC and NHS were taken in amounts to activate 25 mol% of Hep carboxylic groups.

#### 2.4.4. IPECs Characterization

Size and ζ-potential: hydrodynamic diameter, polydispersity index (PDI), and ζ-potential were determined by dynamic light scattering (DLS) and electrophoretic light scattering (ELS), respectively, by using a Zetasizer Nano ZS with He-Ne laser (λ = 633 nm). The 5 wt.% solutions of particles were prepared before DLS measurements from the stock particle suspension. DLS experiments were performed in PBS, pH 7.4, whereas ELS measurements were carried out in deionized water.

Ethidium bromide test: condensing of plasmid DNA (pDNA = pCLuc4) by PLL and the effect of Hep on this interaction was investigated using ethidium bromide (EtBr) intercalation into DNA. Experiments were performed in 96 well plates in 0.1 M PBS, pH 7.4, similarly to [40]. The maximum fluorescence was obtained when EtBr (4 μg/mL) was bound to plasmid DNA (0.6 μg/well) and used as reference. PLL, pDNA, and Hep were added to the wells at different +/− charge ratios. Here and further the number of positive charges was assumed as number of ε-NH_3_^+^ groups of Lys, while the number of negative charges is the number of –PO_4_^−^ groups of DNA, or –SO_3_^−^ groups of Hep. Fluorescence of EtBr was measured using a Varioscan fluorescence plate reader at λ_ex_ = 530 nm and λ_em_ = 590 nm. Two types of experiments with EtBr were performed. At first one, the interaction of PLL and pDNA at different +/− ratios was studied in the absence of Hep in the medium, as well as when Hep was added to the complexes at a 4- and 8-fold −/+ charge excess. At the second experiment, the PLL-pDNA complex was formed at 10-fold +/− charge excess and the displacement of pDNA from the complex in the absence of Hep and with Hep added to give 4- and 8-fold −/+ charge excess was studied.

Agarose gel electrophoresis: complexes were prepared as described above and 24 μL of each complex was loaded to 1% agarose gel in Tris-borate EDTA buffered solution (TBE) pH 8.0. Electrophoresis of the samples was carried out at 100 V during 1.5 h and the gels were stained in EtBr solution (0.5 μg/mL) during 30 min and then imaged. 

Microscale Thermophoresis: PLL was labeled by FITC in Lys-NH_2_/FITC = 100 molar ratio. PLL-pDNA complexes were prepared at constant 50 nM concentration of PLL and serial dilution of the pDNA solution in 0.1 M PBS, pH 7.4, to give 16 different +/− charge ratios of ratios ranging from 17 to 0.001. Samples were incubated for 20 min and loaded into Monolith NT.115 Premium Capillaries. Samples were measured using green excitation and emission with 100% LED and 20% MST power. For determination of Hep effect, the experiment was repeated similarly, but with Hep in each capillary (4-fold −/+ charge excess of Hep over PLL). The analysis of dose-response curves was carried out with GraphPad Prism 8 Software.

#### 2.4.5. Photo-Triggered Linker Decomposition

Linker decomposition in solution by 325 nm laser: 1 mg/mL solution of photosensitive linker in DMSO was exposed to UV laser (λ = 325 nm, power—0.8 and 3 W/cm^2^) as it is presented on Figure S3. The laser beam was vertically directed into the solution of the linker, which was vortexed in 1.5 mL Eppendorf. The exposure times were 5, 15, 30, 45, and 60 min. The quantity of aldehyde (3-nitro-4-formylbenzoic acid) formed was detected via reaction with Schiff’s reagent with application of calibration curve (Figure S4), which was measured with application of commercial reagent. The reaction with Schiff’s reagent was performed as follows. 1 mL of Schiff’s reagent was added to 1 mL of exposed linker solution and the absorbance of colored solution (Figure S5) was measured at λ = 550 nm.

The linker photo-decomposition efficacy (%) was determined by the formula

*Photo-decomposition efficacy* = *Q_laser_*/*Q_lamp_* × 100% (3)

where *Q_laser_* is the amount of 3-nitro-4-formylbenzoic acid formed by irradiation of the linker with a laser; *Q_lamp_* is the amount of 3-nitro-4-formylbenzoic acid formed by irradiating the linker with the source of light which decomposes 100% of linker—wide spectrum UV lamp (power 45 W/cm^2^) for 30 min.

Linker decomposition on the surface of polystyrene particles by 365 nm UV LED lamp: To study the degradation of the linker, it was immobilized on the surface of monodisperse 500 nm polystyrene microparticles (PSt MPs) containing on their surface amino groups. The Boc protection was removed from the primary amino group of the linker and attachment of fluorescent label—NHS-Cy5 was performed. The modified particles were exposed to 365 nm UV LED diode lamp with monochromatic radiation at 365 nm for predetermined time intervals. The quantitative assessment of the degradation of the linker was carried out via measurement of fluorescence intensity of Cy5 in the supernatant. The description of the procedures follows.

250 μL of an aqueous suspension of PSt MPs (10 wt.%, [NH_2_] = 5.8 μmol) was transferred to a 0.01 M bicarbonate buffer solution, pH 9.3 (bb 9.3), via 2 times centrifugation and redispersion at 9000 g during 5 min. The volume of suspension after the procedure was adjusted to 1.0 mL and cooled to 5 °C. Then, 11.4 mg of the linker dissolved in 500 μL of DMSO was added to the suspension. The resulting mixture was stirred on a Vortex at 800 rpm during 1 min. To activate the carboxyl group, 50 mg of dry water-soluble carbodiimide (EDC) was gradually added to the reaction mixture at stirring and left overnight in the dark at 5 °C. Modified particles were cleaned by washing with bb 9.3 as mentioned above.

The precipitate of modified and purified PSt MPs was redispersed using Vortex in a mixture consisting of 0.5 mL of methanol and 0.5 mL of 6 M hydrochloric acid. The Boc deprotection reaction was carried out for 2 h at room temperature. After that, the medium was changed to bb 9.3. To 1.0 mL of the purified suspension 20 μL of Cy5-NHS solution of (0.6 mg/mL, 60 μg, 0.1 μmol) in DMSO was added. The mixture was vortexed and stirred at 450 rpm for 1.5 h at room temperature. The modified PSt MPs were 15 times washed from unbound Cy5-NHS with 0.1 M PBS, pH 7.4, via centrifugation.

To study the linker decomposition 50 μL of modified PSt MPs suspension were exposed to 365 nm UV LED lamp during 1, 10, 20, 40, 60, 100, and 120 min. Then the test tubes were centrifuged at 9000 g for 7 min. The decomposition of the linker was quantified by measuring the fluorescence of Cy5 (λ_ex_ = 646 nm and λ_em_ = 662 nm) in the supernatant using a Varioscan plate reader.

#### 2.4.6. Photo-Triggered pDNA and Oligonucleotide Release

The IPECs with encapsulated pDNA or oligonucleotide and cross-linked with photosensitive linker were prepared as described above. The suspension of the IPECs was exposed to the 365 nm UV Lamp or 325 nm laser similarly to that how it is presented on Figure S3. The exposure time was 30 min. After that the release of pDNA was analyzed by agarose electrophoresis (see description above). The profiles of Cy3-oligo-dT-dA release were measured with application of Eppendorf tubes with filter (30,000 MWCO, Amicon Ultra 0.5 mL, Merck, Darmstadt Germany). The non-cross-linked IPECs, cross-linked IPECs without exposure to UV LED lamp and those after 30 min of exposure were incubated at 37 °C and 1000 rpm. At predetermined time intervals, the tubes were centrifuged at 10,000 g for 15 min. The filtrates were collected and the amount of released oligonucleotide was quantified by fluorescent measurements (λ_ex_ = 550 nm, λ_em_ = 570 nm). 

#### 2.4.7. Cytotoxicity Assay

The viability of HEK 293, BEAS 2B, and NIH-3T3 cells in the presence of different concentrations of linker or IPECs modified with linker was estimated via application of CellTiter-Blue (CTB-assay), which is based on ability of viable cells to reduce non-fluorescent resazurin to fluorescent resorufin. HEK 293 and NIH 3T3 cells were cultivated in Dulbecco’s modified Eagle medium (DMEM) supplemented with 10% (*v/v*) fetal calf serum (FCS) and 1% (*v/v*) penicillin/streptomycin (P/S). BEAS 2B cells were cultured in basal LHC serum-free media supplemented with 10% FCS, and 1% P/S. 8 × 10^3^ cells in 100 μL per well were seeded. After 24 h, this medium was replaced with the culture medium containing tested materials at different concentrations. After 24 or 72 h of cultivation the culture medium was removed and 100 µL of CTB solution (10% stock solution in culture medium) was added to each well and incubated for 2 h. The cells viability was quantified by measuring fluorescence intensity (λ_ex_ = 544, λ_em_ = 590 nm) using a microplate reader. Viability (%) of cells was calculated by comparing the fluorescence signals with control wells containing intact cells. Data are presented as mean ± SD (n = 5).

#### 2.4.8. Particles Cellular Internalization 

Fluorescent microscopy: to study the cellular internalization of IPECs they were covalently labeled by NH_2_-Cy5 probe (1 mol% of Hep –COOH groups). 2 × 10^4^ HEK 293 cells in 300 μL of medium (see Section 2.4.7) were seeded in each well of 8-well glass chamber and cultured for 24 h. Then the incubation medium was removed and 200 μL of medium containing labeled IPECs (50 μg/mL) was added. The cells were incubated during 4 h at 37 °C. After that, the cells were three times washed with warm PBS solution, fixed with PFA (200 μL/well), stained with DAPI (200 μL/well) and imaged by fluorescence microscopy with 20-fold magnification.

Flow cytometry: for evaluation of IPECs cellular internalization dynamics, flow cytometric (FCM) analysis was used. 2 × 10^5^ of HEK 293 cells in 2 mL of medium were seeded in each well of 6-well plates. After 24 h cultivation medium was removed and 1 mL of medium containing labeled IPECs (50 μg/mL) was added. The exposure times were 0, 5, 30, 60, 120, 240, and 360 min. At each time point, the cells were washed with warm PBS, detached by trypsin solution and centrifuged at 300 g for 5 min. Then the cells were washed with PBS and resuspended in 500 µL of PB. The fluorescence signals were measured via flow cytometry. At least 10,000 events per sample were analyzed. Only viable cells were taken for the analysis. Particles cellular internalization is presented as mean fluorescence intensity of viable cells. Data are presented as mean ± SD (n = 5).

#### 2.4.9. GFP Gene Silencing 

NIH 3T3 mouse fibroblast cells producing green fluorescent protein (GFP) were used to test the efficiency of GFP expression knockdown by siRNA molecules delivered by developed IPECs. For this, 2 × 10^3^ cells in 100 μL of medium (see Section 2.4.7) were seeded per each well of a 96-well plate. After 24 h, the medium was removed and 200 μL of PLL-Hep IPECs suspension in culture medium, containing 0.8 μg of encapsulated siRNA was added to each well. In order to study the effect of photo-induced siRNA release some wells with PLL-Hep IPECs cross-linked by photosensitive linker were exposed to 365 nm UV Lamp during 30 min, while other wells were protected by covering with foil. Poly(ethyleneimine) polyplex with the same quantity of siRNA was used as a positive control sample, while untreated cells (without IPECs and not exposed to UV lamp) served as negative control. After 72 h of co-incubation the efficacy of gene silencing was estimated by fluorescence microscopy and flow cytometry, similarly to described above. 

#### 2.4.10. Transfection 

HCE-T cells were cultured in DMEM supplied with 6% FCS, 0.4% P/S, 0.4% l-glutamate, 0.004% epidermal growth factor, 0.053% insulin, 0.2% DMSO, 0.0008% cholera toxin, 0.6% 4-(2-hydroxyethyl)piperasin-1-ethanesulfonic acid. All cells were kept in humidified environment at 37 °C, 5% CO_2_. 5 × 10^4^ cells in 300 μL of medium were seeded in a 48-well plate. After 24 h the medium changed to fresh one and 50 μL of PLL-Hep IPECs containing 1 μg of plasmid DNA (pCLuc4) carrying the firefly luciferase reporter gene were added to each well. To study the effect of photo-induced pDNA release some wells with PLL-Hep IPECs cross-linked by photosensitive linker were exposed to 365 nm UV lamp during 30 min, while other wells were protected by covering with foil. Cells were co-incubated with IPECs during 4 h at 37 °C, then the supernatant was removed and 400 μL of fresh medium was added. After 48 h, the remaining 250 μL of supernatant was collected. The analysis of luciferase expression was carried out in a 96-well plate by measuring luminescence (λ_ex_ = 485 nm and λ_em_ = 535 nm). The transfection efficiency was referred to poly(ethyleneimine)-pDNA polyplex as positive control.

#### 2.4.11. Statistics

The data were expressed as mean (±SD). Statistical analysis and plotting were performed using PRISM software (GraphPad Prism 8.0, La Jolla, CA, USA).

## 3. Results and Discussion

### 3.1. Preparation and Characterization of IPECs

The number of poly-l-lysine with different molecular weights were obtained by l-lysine (Lys) *N*-carboxyanhydride ring-opening polymerization (Table S1). The monomer to initiator ratio variation, allowed to control the molecular weight of polymer product, which is subsequently used to produce the polyplex particles. 

**Figure 1:**
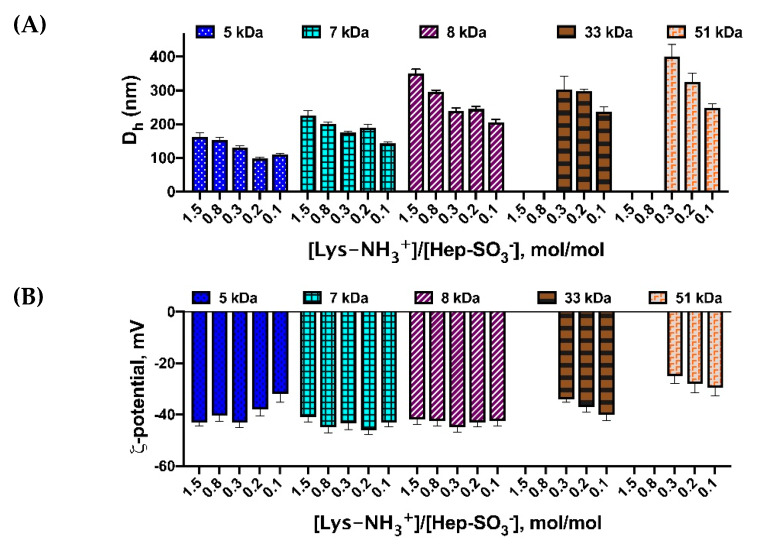
Characteristics of IPECs obtained at different PLL and Hep charged groups molar ratios and with application of PLL of different molecular weights: (**A**) particles hydrodynamic diameter; (**B**) particles ζ-potential.

PLL-Hep particles were prepared at different ratios of PLL and Hep and using PLL of different molecular weights. The dependences of particle size on PLL molecular weight and the molar ratio of PLL and Hep charged units, namely, –NH_3_^+^ groups of Lys and SO_3_^−^ groups of Hep, are shown in Figure 1. Taking into account that about 60% of Hep units contain three sulfo-groups (IdoA2SO_3_-GlcNSO_3_6SO_3_), while other minor part contains mono-sulfated (GIcA) and non-sulfated (GIcNAc) saccharide units [43], we have assumed that each averaged unit of Hep contains two sulfo-groups. Based on this approximation we have studied the effect of charge ratios and PLL molecular weight on the size and ζ-potential of resulted particles (Figure 1). 

One can observe that particle size (Figure 1A) decreases when PLL with smaller molecular weight is applied. Moreover, the *D_h_* of obtained IPECs is smaller when greater quantity of Hep is applied. The effect of PLL molecular weight could be explained by the accessibility of Lys–NH_3_^+^ for interaction. When molecular weight of PLL is growing, some charged groups could be hindered by the polymer chain conformation and prevent ionic interaction. Moreover, PLL with higher molecular weight possess elevated viscosity, which also complicate the formation of IPEC. In addition, it was shown (Figure 1A) that the higher the concentration of heparin is, the more compact structures are formed. It is likely that an increase in the concentration of heparin within the applied range led to an increase in the number of both positive (PLL-Hep) and negative (Hep-Hep) electrostatic interactions, which contributed to the compaction of particles and a decrease in their aggregation, correspondingly.

ζ-potential of the particles obtained was measured to check the stability of systems. The presented results demonstrate (Figure 1B) that regardless of the applied charge ratios all prepared particles possess the negative surface charge. This could be associated with the predominantly surface arrangement of Hep within the particles. Based on these results, we have applied Hep excess and PLL with molecular weight of 5000 in all further experiments.

**Figure 2:**
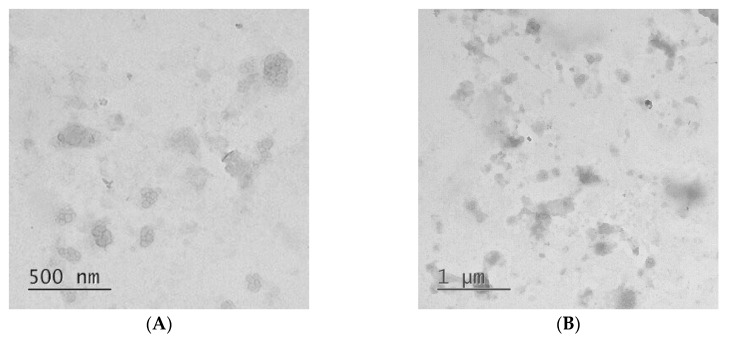
Images of PLL-Hep IPECs (ratio [NH_3_^+^]:[SO_3_^−^] = 1:3 moL/moL]) obtained by transmission electron microscopy: (**A**)—500 nm scale; (**B**)—1000 nm scale.

The morphology of the particles was studied by transmission electron microscopy (TEM, Figure 2). One can observe that formed IPECs possess the spherical shape and the size around 100 nm: the average size (n = 50) of nanoparticle was calculated to be 92 ± 11 nm. The tendency to the formation of small aggregated nanostructures was also detected.

### 3.2. Hep Effect on PLL Interaction with Genetic Constructions

It is known that PLL interaction with polynucleotides (DNA, RNA) leads to the formation of stable polyplexes [20]. Also, it was estimated previously [40] that such interaction is affected by a presence of negatively charged glycosaminoglycans such as Hep. Here, we have quantified the effect of Hep on the PLL-pDNA interactions by using different methods. In this study, we have applied pCMVL plasmid DNA (pDNA) as a model. 

**Figure 3:**
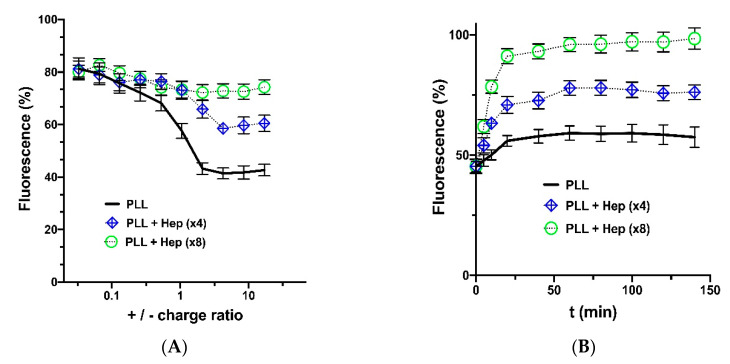
Ethidium bromide test: (**A**) the effect of Hep on condensation of pDNA by PLL (+/− charge ratio is determined by molar ratio of Lys–NH_3_^+^ groups and PO_4_^−^ groups of pDNA). Hep was taken in quantities to obtain 4- and 8-fold molar excess of SO_3_^−^ groups over Lys–NH_3_^+^; (**B**) displacement of pDNA from the IPECs with PLL after addition of the Hep excess obtained at different PLL/Hep ratios. The values are expressed as a percentage of the ethidium bromide fluorescence without the carrier. Each value is the mean of triplicates ± SD.

The effect of Hep on the polycation-DNA interaction has been demonstrated by addition of ethidium bromide (EtBr) in the system [40]. This dye is known to show greater fluorescence in complex with DNA. The ongoing condensation of DNA with polycation results in partial displacement of ethidium bromide from DNA causing a decrease in the fluorescence signal at 590 nm. Negatively charged Hep displaces cationic carrier from DNA and allows for binding of ethidium bromide to DNA. One can observe (Figure 3A) that DNA condensation by PLL in the medium containing Hep is hindered even at significant excess of PLL over pDNA. Also, it was shown that the addition of Hep excess can displace DNA from its complex with PLL (Figure 3B), resulting in DNA release. These observations were also in a good agreement with the data obtained by agarose gel electrophoresis, according to which DNA was not mobile in the IPEC with PLL, but gains certain mobility after the addition of Hep (Figure S6).

**Figure 4:**
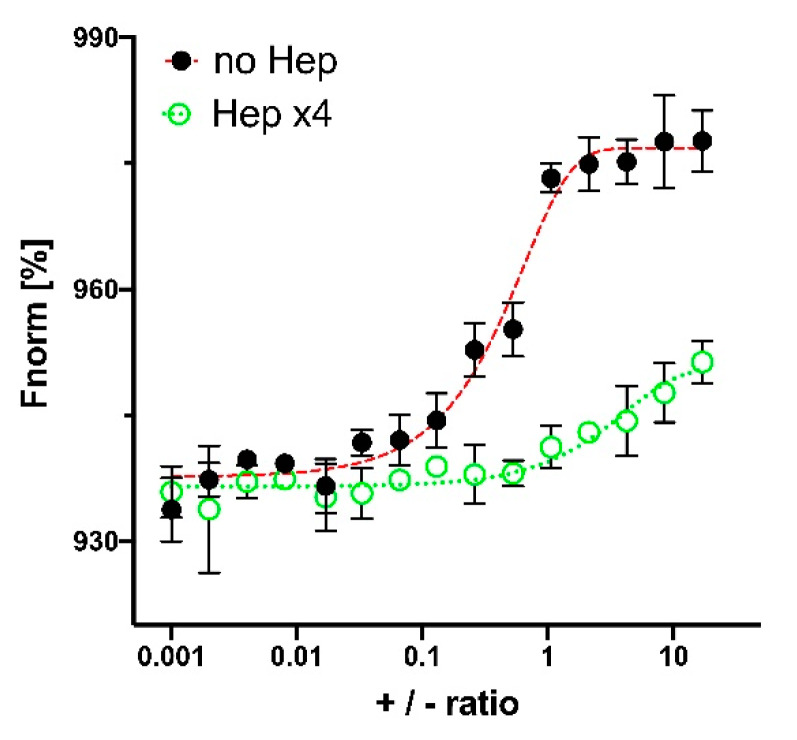
Effect of Hep excess on PLL-pDNA interaction studied by microscale thermophoresis method. +/− charge ratio is determined by molar ratio of Lys–NH_3_^+^ groups and PO_4_^−^ groups of pDNA. Red line—interaction of PLL with pDNA. Green line—interaction of PLL with pDNA in the medium containing Hep excess over PLL ([SO_3_^−^]/[Lys–NH_3_^+^] = 4). PLL-FITC conjugate was used as target, which was titrated by ligand (pDNA = pCMVL) different concentrations.

The effect of Hep on PLL-DNA interaction was also studied by microscale thermophoresis. FITC-labeled PLL was applied as target molecule and pDNA served as a ligand, the concentration of which was varied to give different +/− ratios. The interaction between PLL and DNA in the absence of Hep was characterized by typical binding curve, while PLL–DNA interaction was obviously shielded by addition of Hep (Figure 4).

The determined effect of Hep on release of pDNA from IPEC with PLL allowed us the proposal of the possibility to assemble the construction for triggered release. In our supposition such system could be based on PLL-pDNA polyplex covered by excess of Hep. The formed complex could be stabilized by special photosensitive cross-linker, which prevents the polyelectrolytes movement and detachment. After action receiving the photon the decomposition of the linker allows the molecular movement and macromolecules of Hep should displace the DNA form the complex.

### 3.3. Assembling of Photosensitive IPECs and Linker Photo-Decomposition 

In order to create IPECs capable of triggered release of loaded genetic construction under the action of optical irradiation with specific wavelength, it was necessary to obtain the particles containing in their structure the molecules of photosensitive linker. Among other linkers, 4-bromomethyl-3-nitrobenzoic acid and its derivatives are widely used due to their ability to photo-decomposition when subjected to UV radiation in the wavelength range from 300 to 400 nm [44]. 

**Figure 5:**
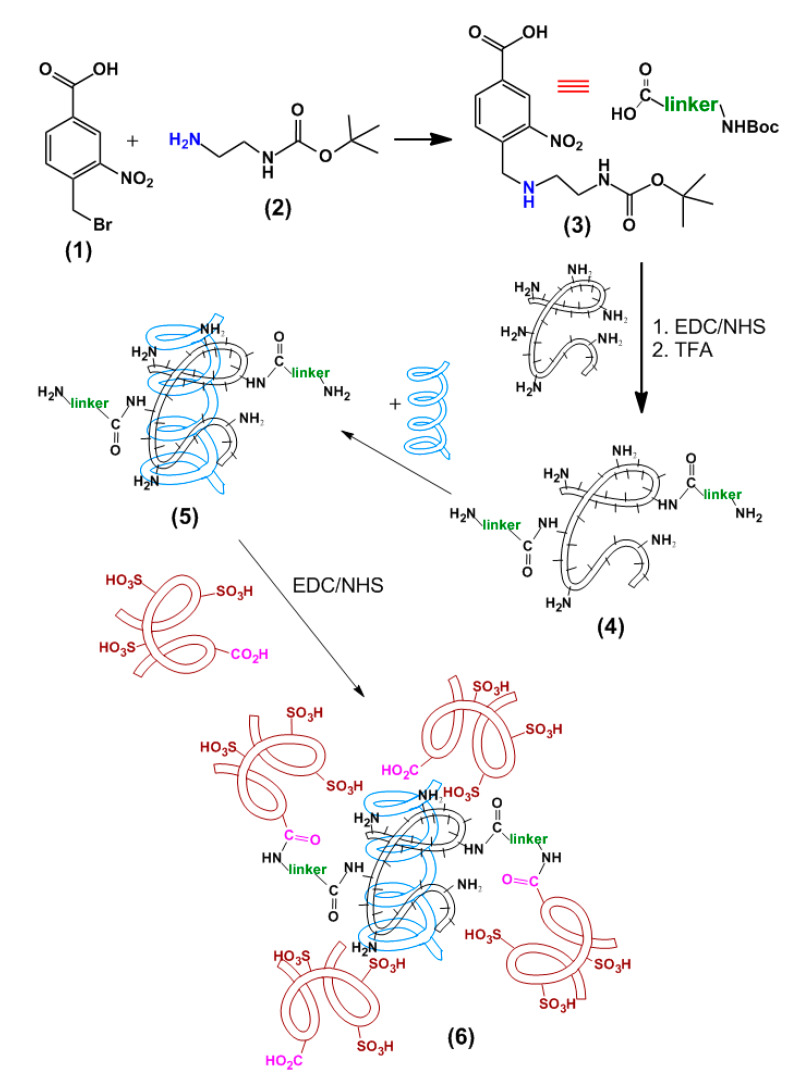
Assembling of photosensitive IPECs by cross-linking with photosensitive linker.

In the current study, we have proposed cross-linking of IPEC components—namely, PLL and Hep—by photosensitive linker as a strategy for triggered release of pDNA or siRNA (Figure 5). To obtain the linker capable of the reaction both with amino groups of PLL and carboxylic groups of Hep, 4-bromomethyl-3-nitrobenzoic acid (Figure 5, compound **1**) was modified by mono-Boc-protected ethylenediamine (Figure 5, compound **2**) via the reaction of bromine nucleophilic substitution. The carboxylic group of the compound (Figure 5, compound **3**) was activated by carbodiimide and *N*-hydroxysuccinimide to react with PLL ε-amino groups. The conjugate obtained (Figure 5, compound **4**) was subjected to deprotection and the modified polycation was used to condensate genetic construction (Figure 5, compound **5**). After addition of Hep the carbodiimide chemistry was applied again to form small amount of covalent bonds between linker amino groups and –CO_2_H groups of Hep (Figure 5, compound **6**). Here we suppose only minor participation of Lys ε-NH_2_ groups in such covalent interactions due to their predominant participation in ionic interactions with DNA. 

The structure of compound **3**, which will be further referred as ‘linker’, was confirmed by ^1^H NMR (Figure S7). Before the application of a linker for assembling of photosensitive nanogels it was interesting to study photo-decomposition of the linker molecule. According to the previous studies [45] the photo-induced decomposition of a linker should result in the formation of aldehyde (Figure S8), which could be detected via 1H NMR (Figure S9) and quantified by the reaction with aldehyde-specific Schiff’s reagent (Figure S5).

**Figure 6:**
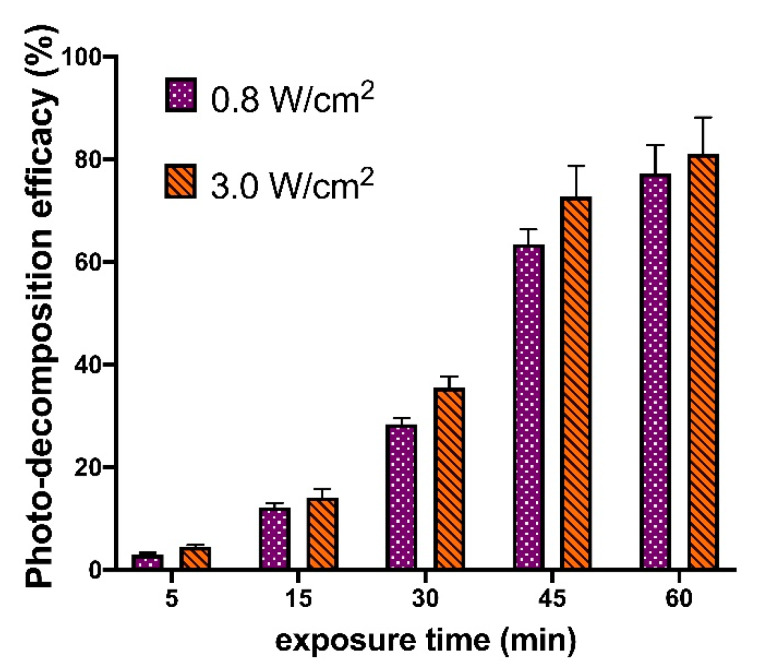
Effect of irradiation time and laser power on the efficacy of 325 nm laser photo-induced linker decomposition in DMSO solution.

The effects of laser power and time of exposure on the efficacy of linker decomposition were studied (Figure 6). For that, the laser with 3 W/cm^2^ and 0.8 W/cm^2^ power was applied to the samples for 5, 15, 30, 45, and 60 min. The linker solutions irradiated for 30 min by UV lamp with a power of 45 W/m^2^ were assumed to contain only the products of decomposition and were used as positive control.

The results obtained showed very close photo-decomposition efficacies for the lasers with different power (Figure 6). The same data allow conclusion that the applied 325 nm laser leads to the decomposition of approximately 40% linker in a solution after 30 min and nearly the complete decomposition could be attained within 1 h.

**Figure 7:**
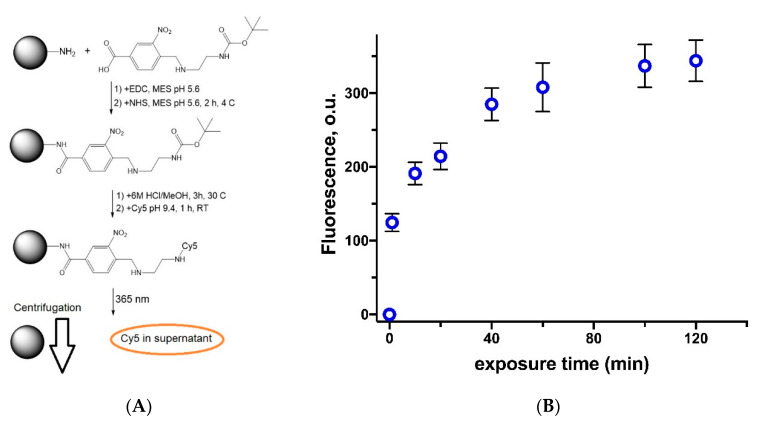
Study of linker decomposition after exposure to 5W LED UV lamp 365 nm: (**A**) the scheme of experiment: linker immobilization on the surface of polystyrene microparticles, Cy5 fluorescent probe attachment, exposure to the lamp and separation of microparticles by centrifugation; (**B**) the effect of polystyrene microparticles with immobilized linker exposure time on the fluorescence of released Cy5 probe in the supernatant. Conditions: 0.1 M PBS, pH 7.4; microparticles suspension concentration—5 wt.%.

Another approach to the study of photo-induced decomposition of studied linker (Figure 5, compound **3**) consisted in its covalent immobilization on the surface of 500 nm monodisperse polystyrene (PSt) microspheres, bearing NH_2_ groups on the surface (Figure 7A). After deprotection of Boc the free amino groups of a linker were used for coupling of NHS-Cy5 fluorescent probes. The particle suspension was subjected to the 5W LED UV lamp 365 nm irradiation. The efficiency of linker decomposition was quantified by measurement of Cy5 probe fluorescence in the supernatant after the separation of particles by centrifugation (Figure 7B). The sudden growth of fluorescence in the first minutes of exposure was observed. It is worth noting that after 80 min of exposure no fluorescence gain was detected, so decomposition of a linker could be considered to be complete.

Thus, we have shown that suggested linker could be photo-chemically decomposed under the exposure to 325 nm laser or LED lamp with the wavelength of 365 nm. The decomposition starts at first minutes but reaches the plateau after about 60–80 min of exposure. 

As was mentioned above, the linker should be bound to PLL macromolecules prior to addition of genetic construction and polyplex formation. The amount of linker attached to PLL was determined by the measurement of linker quantity in a supernatant. Furthermore, the amino group of a linker was deprotected and the complex with model Cy3-labeled 23-base pairs duplex of oligothymidine and oligoadenine (oligo-dT-dA) was obtained. Then the EDC/NHS activated Hep was added to finalize the nanogel assembling (Figure 5). The nanogels were obtained at different PLL:Hep:linker ratios and the effect of particles’ cross-linking on their size and ζ-potential was evaluated (Table 1). It was shown that the all linker introduced into reaction was bound when an excess of PLL amino groups was applied. According to presented data, the size and ζ-potential of cross-linked by photosensitive linker IPECs were not affected by such modification (compare the data in Table 1 and Figure 1).

The study of IPECs stability over two weeks showed that the cross-linking of particles stabilizes their size, while ζ-potential stay nearly unchanged for both cross-linked and non-cross-linked systems (Figure S10). 

### 3.4. Photosensitive Release 

**Figure 8:**
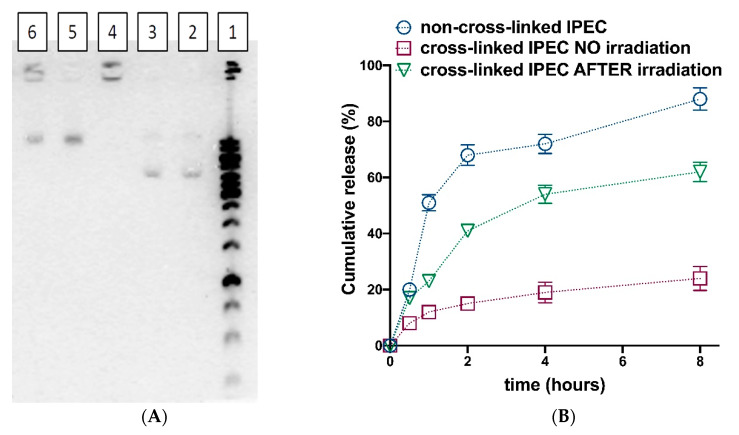
Photosensitive release studies: (**A**) Release of pDNA (pCLuc4) detection via agarose gel electrophoresis: (1) ladder; (2,3) pDNA; (4) pDNA in cross-linked IPECs ([Lys–NH_3_^+^]:[pDNA–PO_4_^−^]:[Hep–SO_3_^−^]:[linker] = 1:0.5:3:0.8) without UV treatment; (5) pDNA in cross-linked IPECs ([Lys–NH_3_^+^]:[pDNA–PO_4_^−^]:[Hep–SO_3_^−^]:[linker] = 1:0.5:3:0.8) after 30 min exposure to 5W UV 365 nm LED lamp; (6) pDNA in cross-linked IPECs ([Lys–NH_3_^+^]:[pDNA–PO_4_^−^]:[Hep–SO_3_^−^]:[linker] = 1:0.5:3:1.6) after 30 min exposure to 5W UV 365 nm LED lamp. (**B**) Release of Cy3-oligo-dT-dA from IPECs (non-cross-linked, cross-linked without further treatment and cross-linked with subsequent 30 min exposure to 5W UV 365 nm LED lamp) evaluated via measurement of fluorescence of filtrate after separation with ultrafiltration (MWCO 3000). The oligo-dT-dA containing IPEC was obtained at following ratios: [Lys–NH_3_^+^]:[oligo-dT-dA-PO_4_^−^]:[Hep–SO_3_^−^]:[linker] = 1:1:3:1.6.

The possibility of photosensitive release was studied with application of pDNA (pCLuc4) and Cy3 labeled oligo-dT-dA (Figure 8). It should be noted that the encapsulation efficacy for both pDNA and oligo-dT-dA at chosen ratios was equal to 100%. The agarose electrophoresis showed no mobility of pDNA when it was encapsulated into the complexes, which were subsequently cross-linked by photosensitive linker (Figure 8A, lane 4). The exposure to 5W UV 365 nm LED lamp led to the appearance of the pDNA bands in the gel (Figure 8A, lanes 5 and 6). It is worth noting that when higher linker quantity was applied (Figure 7A, lane 6) some amount of pDNA seemed to stay immobilized into the IPEC. This could be caused by the incomplete linker decomposition, or by the increased amount of non-regiospecific cross-links formation (between amino and carboxylic groups of Lys and Hep, correspondingly) because of elevated number of activating agents added in this case.

The release of Cy3-labeled oligo-dT-dA, which served as physico-chemical model of siRNA, was studied using ultrafiltration of IPECs colloid solutions and subsequent measuring of filtrate fluorescence (Figure 8B). The release of oligo-dT-dA from non-cross-linked IPEC was fast. This is in a good agreement with previous observations (Figure 3). Cross-linking IPEC according to the proposed strategy led to only minor oligo-dT-dA cumulative release values. After exposure to 5W UV 365 nm LED lamp during 30 min the IPEC retrieves the ability to release the complexed oligo-dT-dA (Figure 8B). 

The presented data allowed us to conclude that the proposed method of light-sensitive IPECs construction could give the systems, which could release the entrapped genetic constructions upon exposure to the source of electromagnetic radiation with certain wavelength. It should be noted that results obtained after exposure of IPECs to 325 nm laser and UV 365 nm LED lamp were nearly the same. Thus, we have applied the UV 365 nm LED lamp in further experiments with cells.

### 3.5. Cytotoxicity and Cellular Internalization

**Figure 9:**
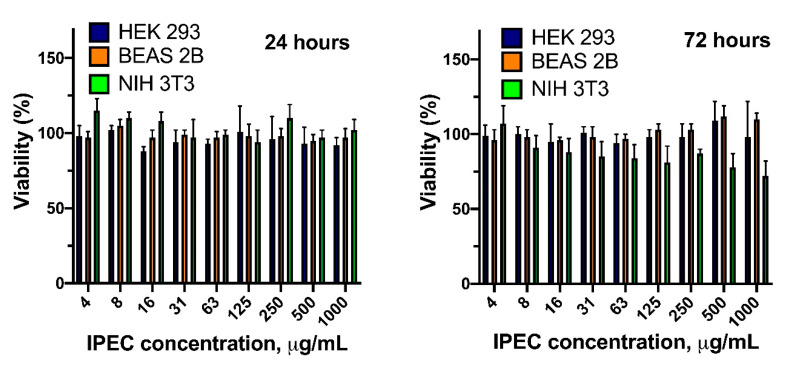
CTB test: viability of HEK 293, BEAS 2B and NIH 3T3 cells incubated with different concentrations of cross-linked IPECs ([Lys–NH_3_^+^]:[Hep–SO_3_^−^]:[linker] = 1: 3:0.8).

In order to test the possibility of obtained systems application for transfection of genetic constructions, the cross-linked IPECs effect on cells viability was evaluated. Despite the slight dose dependent cytotoxicity of the linker molecule at the concentrations above 125 μg/mL (Figure S11), we have found no negative effect of cross-linked IPECs on a viability of three different types of cell lines (Figure 9). This result allowed proposal of the systems obtained for application as transfection agents.

An important feature of the system to be used in transfection is the ability to internalize into the cells. We have applied flow-cytometry to analyze the penetration of IPECs into HEK 293 cells. In order to visualize the assembled IPECs in the cells, they were modified by amino-Cy5 probe (Figure S12) while the cells were stained by DAPI. 

**Figure 10:**
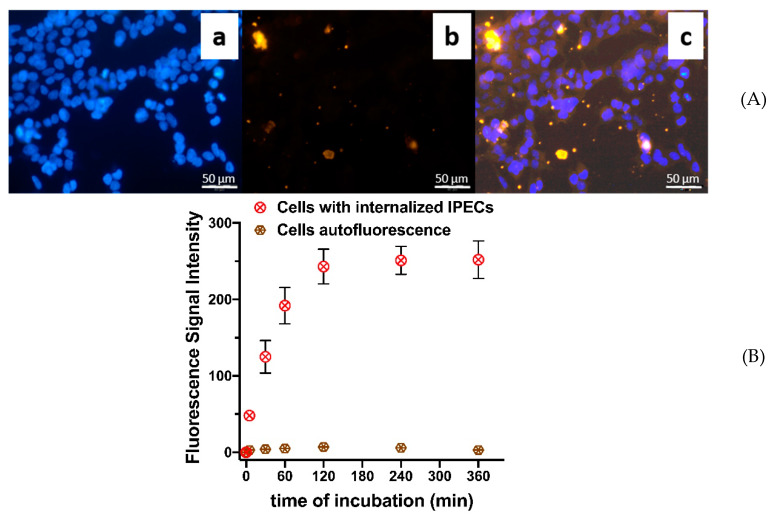
IPECs cellular internalization studies: (**A**) Visualization of IPECs cellular internalization by fluorescent microscopy: **a**—DAPI-stained cells; **b**—Cy5-labeled IPECs; **c**—merging of a and b. Time of co-incubation—2 h; (**B**) Fluorescence signal intensity after incubation of HEK 293 cells with cross-linked IPEC ([Lys–NH_3_^+^]:[Hep–SO_3_^−^]:[linker] = 1:3:0.8). The IPEC concentration—0.1 mg/mL. The data are given as mean ± SD (n = 5).

The IPECs cellular penetration was visualized by fluorescence microscopy (Figure 10A). The particles and their aggregates can be observed within the cytoplasm of the cells revealing the successful penetration of obtained IPECs into HEK 293 cells.

The quantitative estimation of IPECs cellular internalization was carried out with the application of flow cytometry (Figure 10B). One can observe that the particles are quite effectively captured by the cells during first hour of co-incubation and the internalization process reaches the plateau within about 2 h. Interestingly, we have observed quite intensive cellular penetration of particulate formulations, which possess negative ζ-potential. However, this is in line with our recent results on cellular internalization of PLL and Hep covering poly(lactic acid)-based nanoparticles [46].

### 3.6. GFP Gene Expression Knockdown

**Figure 11:**
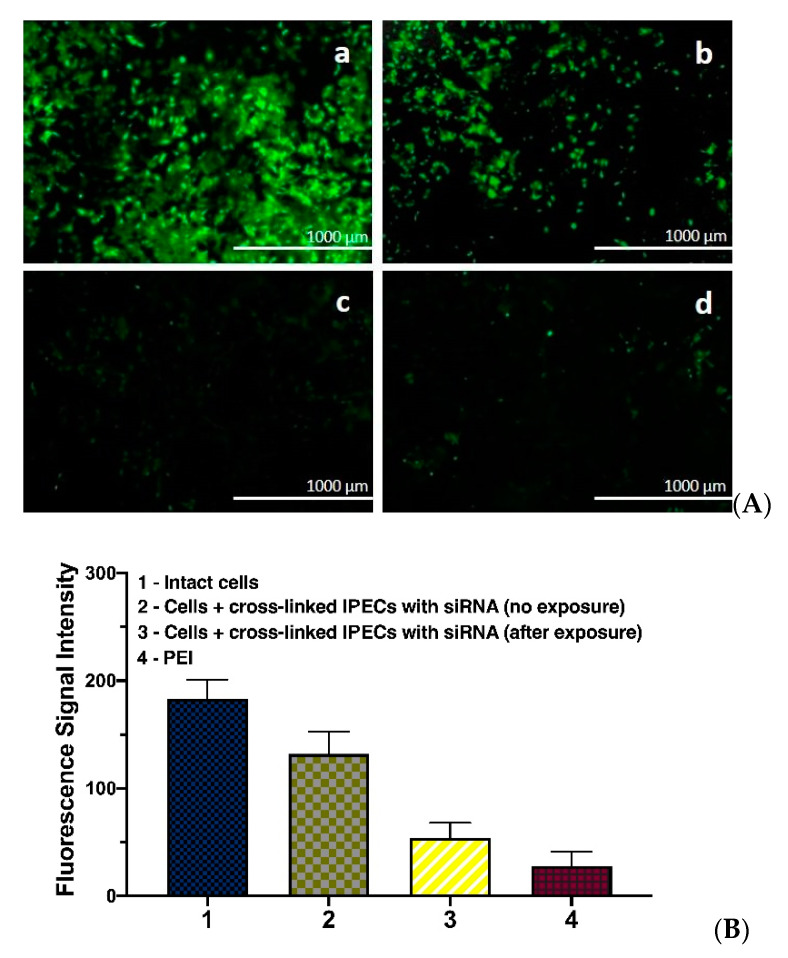
RNA interference by anti-GFP small interfering RNA that was delivered by PLL/Hep IPECs ([Lys–NH_3_^+^]:[siRNA–PO_4_^−^]:[Hep–SO_3_^−^]:[linker] = 1:1:4:0.8) in NIH-3T3 cell line. (**A**) Fluorescence microscopy images of NIH-3T3 cells after transfection with anti-GFP siRNA complexed by IPECs: **a**—intact cells; **b**—cells incubated with cross-linked anti-GFP-siRNA/IPECs; **c**—cells incubated with cross-linked anti-GFP-siRNA/IPECs and subjected to the treatment 5W UV 365 nm LED lamp for 30 min; **d**—cells incubated with anti-GFP-siRNA/PEI polyplex. (**B**) Flow cytometry detection of GFP fluorescence after co-incubation with anti-GFP siRNA containing complexes. Co-incubation time was 4 h. Mean (± SD), n = 5.

The small interfering RNA (siRNA) technology is one of the perspective medical treatments, which allows the knockdown of the expression of pathology causing proteins. In order to test the potential of systems obtained in this study, we have prepared PLL/Hep IP-ECs, containing siRNA ([Lys–NH_3_^+^]:[siRNA–PO_4_^−^]:[Hep–SO_3_^−^]:[linker] = 1:1:4:0.8), which could knockdown the expression of green fluorescent protein (GFP). The IPECs were cross-linked by a photosensitive linker according to the procedure described above and applied for anti-GFP siRNA delivery into expressing GFP mouse fibroblast cells (NIH-3T3). The knock-down of GFP expression was analyzed by fluorescence microscopy and flow cytometry (Figure 11). The intact cells and PEI-mediated siRNA delivery were applied as negative and positive controls, respectively. 

The delivery of siRNA by cross-linked IPECs have resulted in minor decrease of GFP fluorescence (Figure 11A, b and Figure 11B, 2). When the suspension of the cells with co-incubated cross-linked IPECs was treated by 5W UV 365 nm LED lamp for 30 min before attachment, the GFP expression was significantly decreased (Figure 11A, c and Figure 11B, 3) in comparison to that in intact cells (Figure 11A, a and Figure 11B, 1). Notable, that confluence of cells, their attachment and shape were minimally affected by the UV irradiation (Figure S13). However, further studies should be made to evaluate the effect of such treatment on the cells fate and behavior. The result obtained with application of developed IPECs is slightly worse than that in the case of PEI-mediated delivery of siRNA. This points out the necessity of further optimization of the system for better delivery and release of genetic materials. Nevertheless, the possibility and the effect of photosensitive siRNA release with the developed systems was demonstrated.

### 3.7. Transfection Efficacy

**Figure 12:**
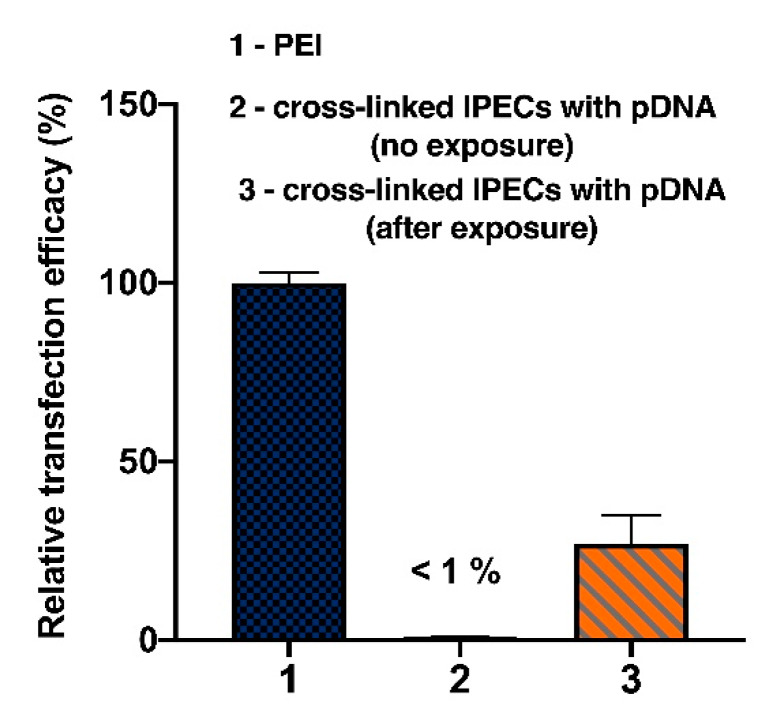
Transfection of HCE-T cells with pCMVL by IPECs ([Lys–NH_3_^+^]:[pDNA–PO_4_^−^]:[Hep–SO_3_^−^]:[linker] = 1:0.5:4:0.8). Mean (±SD), n = 5.

The ability of IPECs obtained to light-sensitive delivery of genes to living cells was studied with the application of plasmid DNA (pCLuc4), which contained the firefly luciferase gene. Human corneal epithelial cells (HCE-T) were incubated with IPECs ([Lys–NH_3_^+^]:[pDNA–PO_4_^−^]:[Hep–SO_3_^−^]:[linker] = 1:0.5:4:0.8). Similar to the siRNA delivery, the effect of treatment with 365 nm LED lamp on the transfection efficacy was evaluated (Figure 12). The PEI mediated transfection was carried out as positive control. Cross-linked IPECs showed no ability to transfect the cells without exposure to the source of light. At the same time, the exposure of the cells to the 365 nm LED lamp led to the appearance of luciferase fluorescence. The efficacy of transfection in this case was 27% with respect to PEI mediated transfection. Thus, the light activation of pDNA transfection was shown for the developed systems.

## 4. Conclusions

In the present work, the biocompatible poly-l-lysine/heparin interpolyelectrolyte complexes were used to prepare the systems capable of photosensitive release. It was shown that such complexes have to contain the excess of heparin over the poly-l-lysine in order to displace the genetic construction from the polyplex. The triggered release of genetic compounds from IPECs was provided with the use of special photosensitive linker based on 4-bromomethyl-3-nitrobenzoic acid. The carboxylic group of this linker was used for conjugation with poly-l-lysine, while its amino group was involved in the reaction with heparin covering the polyplex. The photo-induced decomposition of the linker, as well as triggered release of model plasmid DNA and oligonucleotide, were testified. The assembled photosensitive interpolyelectrolyte complexes were capable of cellular internalization. Also, the structures obtained were shown to be prospective for photosensitive protein expression knockdown and transfection of plasmid DNA. However, due to hardness of UV irradiation and its possible negative effect on the cells the NIR light sensitive linkers should be further investigated for the developed systems implementation.

## Figures and Tables

**Table 1:** Characteristics of IPECs obtained with application of photosensitive linker. The complex of PLL with oligo-dT-dA was formed prior to addition of Hep. [Lys–NH_3_^+^]:[olig-dT-dA-PO_4_^−^] ratio was 1:0.5 in all cases.

#	[Lys–NH_3_^+^]:[Hep–SO_3_^−^]:[Linker] mol/mol/mol	*q* (Bound Linker), mol%	*D_h_*, nm	ζ-Potential, mV	PDI
1	1:3:0.4	100	112 ± 5	−38 ± 1	0.2
2	1:3:0.8	98	105 ± 2	−31 ± 2	0.2
3	1:3:1.6	81	103 ± 1	−38 ± 1	0.1

## Supplementary Materials

The following are available online at https://www.mdpi.com/2073-4360/12/5/1077/s1. Figure S1: Calibration curve showing the dependence of the fluorescence intensity (λ_ex_ = 550 nm, λ_em_ = 570 nm) of the solution on the concentration of Cy3-oligo-dT-dA, plotted to determine the amount of encapsulated oligonucleotide as well as to quantify its release; Figure S2: A calibration curve showing the linear dependence of the optical density of the solution on the concentration of the photosensitive linker. The curve was plotted to determine the content of unbound linker upon conjugation with PLL. The absorption was measured at a wavelength of 325 nm. The molar extinction coefficient was estimated as ε = 619 L·mol^−1^·cm^−1^; Figure S3: The scheme of the installation for irradiation of IPECs with a 325 nm laser; Figure S4: Calibration curve showing the dependence of the optical density of the solution on the concentration of 3-nitro-4-formylbenzoic acid, plotted to determine the amount of released decomposition product, which entered into a qualitative reaction with Schiff’s reagent. The absorption was measured at a wavelength of 550 nm. The molar extinction coefficient was estimated to be ε = 1521 L·mol^−1^·cm^−1^; Figure S5: Reaction of linker decomposition aldehyde product with Schiff’s reagent (A); colour reaction with Schiff’s reagent, used for photo-colormetric quantification of the reaction; Figure S6: Agarose gel electrophoresis: (1)—ladder; (2,3)—pDNA; (4)—pLys+pDNA; (5)—pLys+pDNA+4xHep; (6)—pLys+pDNA+8xHep; Figure S7: The ^1^H NMR spectrum of 4-(((2-((tert-butoxycarbonyl)amino) ethyl)amino)methyl)-3-nitrobenzoic acid; Figure S8: Linker (4-(((2-((tert-butoxycarbonyl)amino)ethyl)amino)methyl)-3-nitrobenzoic acid decomposition under UV radiation at λ = 325 nm; Figure S9: Differences in the ^1^H NMR spectra of the initial linker (A,C) and its photodestruction product (B,D) when irradiated with a laser with a wavelength of 325 nm with a power of 3 W/cm^2^ for 15 min. The signal of aldehyde (B) and hemiacetal (D) groups were detected. Spectra were obtained in DMSO-d6; Figure S10: Stability of IPECS: particles hydrodynamic diameter (A) and ζ-potential (B) change with time. Conditions: 0.1 M PBS buffer solution, pH 7.4; particles concentration 0.1 mg/mL, 37 °C; Figure S11: CTB test: viability of HEK 293 and BEAS 2B cells incubated with different concentrations of photosensitive linker (see Figure 5, compound **3**); Figure S12: Modification of IPECs by amino-Cy5 probe; Figure S13: Optical microscopy: mouse fibroblast cells (NIH-3T3) morphology after exposure to 325 nm laser; Table S1: The effect of [Monomer]:[Initiator] molar ratio on the M_n_ of obtained PLL.
