# Supplementary material for: Photosensitive Poly-l-lysine/Heparin Interpolyelectrolyte Complexes for Delivery of Genetic Drugs
Source: Polymers (Basel). 2020 May 8;12(5):1077. doi: 10.3390/polym12051077 (PMC7285230; doi:10.3390/polym12051077)
Supplement: Supplementary file 1 [file polymers-12-01077-s001.pdf]

polymers

Supporting information

Photo-sensitive poly-L-lysine/heparin interpolyelectrolyte complexes for delivery of genetic drugs

Viktor Korzhikov-Vlakh[1*], Iuliia Katernuk[1], Antonina Lavrentieva[2], Ivan Guryanov[1], Vladimir Sharoyko[1], Alina A. Manshina[1], Tatiana B. Tennikova[1]

[1] Saint Petersburg State University, Institute of Chemistry, St. Petersburg, Peterhoff, 198504, Universitetskii pr. 26, Russia; v.korzhikov-vlakh@spbu.ru (V.K.-V.); katernuk02@gmail.com (I.K.); ivan.guryanov1@gmail.com (I.G.); sharoyko@gmail.com (V.S.); a.manshina@spbu.ru (A.A.M.); tennikova@mail.ru (T.B.T.)

[2] Institute of Technical Chemistry, Leibniz University, Callinstrasse 5, 30167, Hannover, Germany; lavrentieva@iftc.uni-hannover.de (A.L.)

* Correspondence: v.korzhikov-vlakh@spbu.ru; Tel.: +7-(921)-420-8127

Table S1. The effect of [Monomer]:[Initiator] molar ratio on the M_n of obtained PLL. *Conditions: Monomer (NCA of Lys) concentration – 4 wt.%; initiation with n-hexylamine; T = 25 °C; reaction in 1,4-dioxane during 48 hours. The M_n was determined by GPC in DMF for polymers prior to deprotection with application of PMMA standards (10 – 50×10^3 g/mol).*

Sample #	[M]/[I], mol/mol	M_n	Đ
1	250	51000	1.4
2	200	33000	1.3
3	150	8000	2.5
4	100	7000	1.8
5	50	5000	1.6

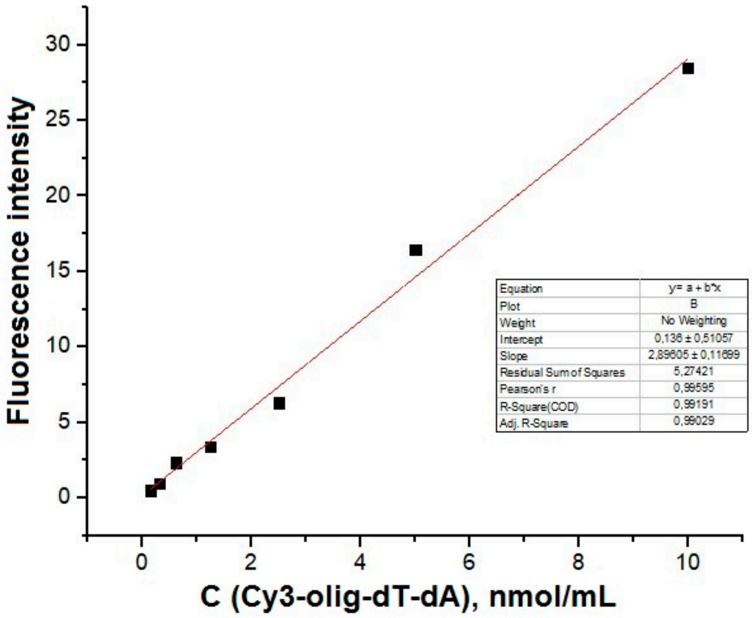

Figure S1. Calibration curve showing the dependence of the fluorescence intensity (λex = 550 nm, λem = 570 nm) of the solution on the concentration of Cy3-oligo-dT-dA, plotted to determine the amount of encapsulated oligonucleotide as well as to quantify its release.

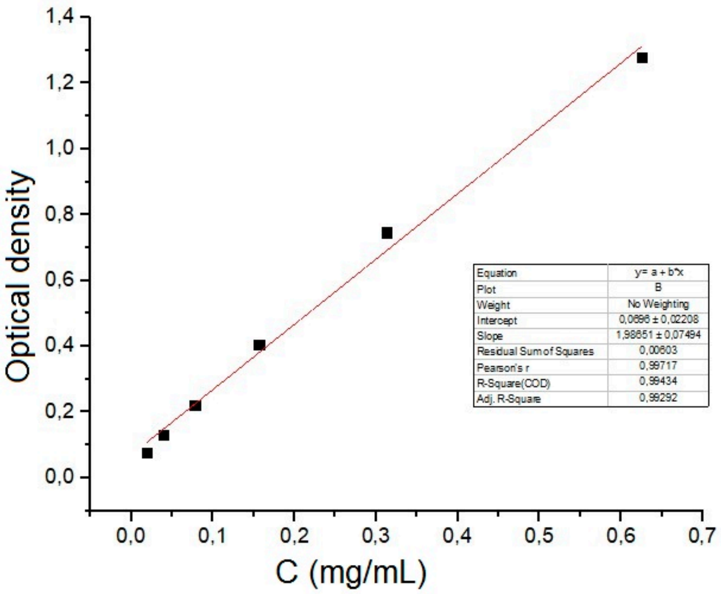

Figure S2. A calibration curve showing the linear dependence of the optical density of the solution on the concentration of the photo-sensitive linker. The curve was plotted to determine the content of unbound linker upon conjugation with PLL. The absorption was measured at a wavelength of 325 nm. The molar extinction coefficient was estimated as ε = 619 L mol^{-1} cm^{-1}.

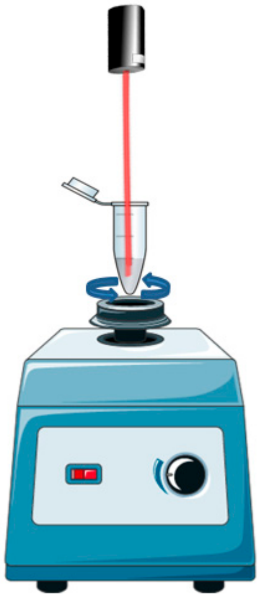

Figure S3. The scheme of the installation for irradiation of IPECs with a 325 nm laser.

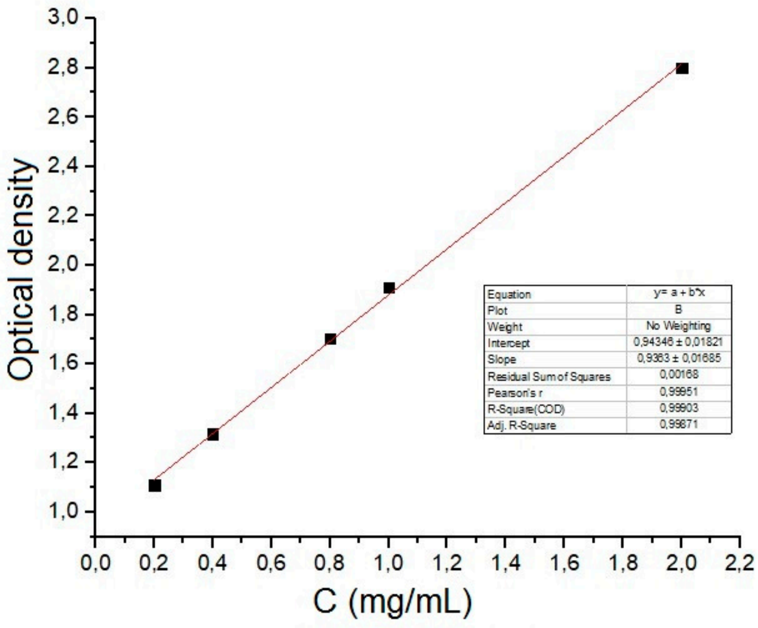

Figure S4. Calibration curve showing the dependence of the optical density of the solution on the concentration of 3-nitro-4-formylbenzoic acid, plotted to determine the amount of released decomposition product, which entered into a qualitative reaction with Schiff's reagent. The absorption was measured at a wavelength of 550 nm. The molar extinction coefficient was estimated to be $\varepsilon = 1521\ L\ mol^{-1}\ cm^{-1}$.

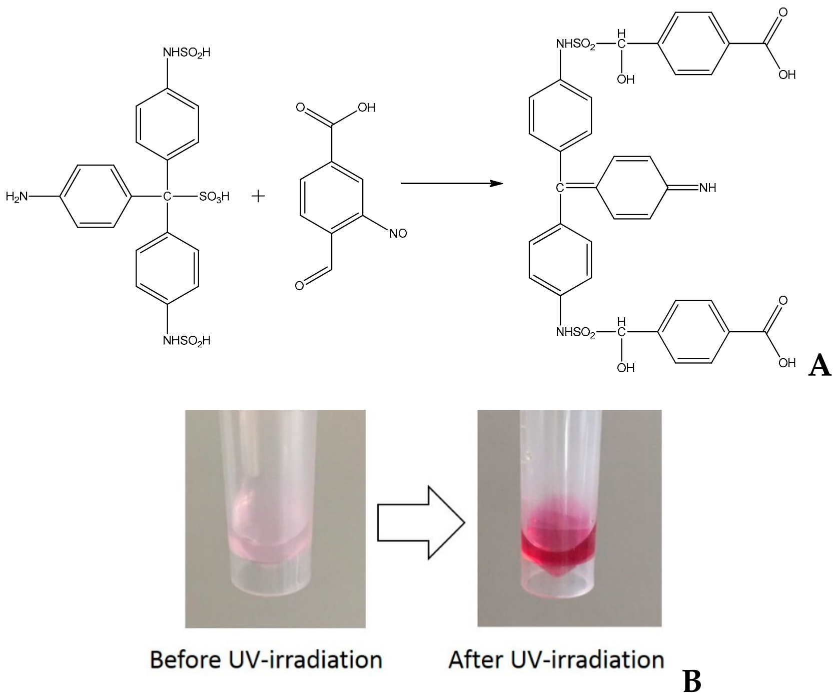

Figure S5. Reaction of linker decomposition aldehyde product with Schiff's reagent (A); colour reaction with Schiff's reagent, used for photo-colormetric quantification of the reaction.

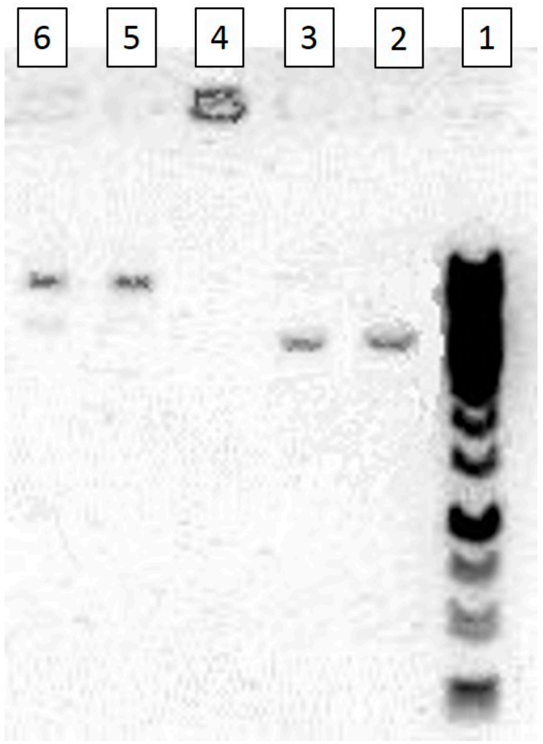

Figure S6. Agarose gel electrophoresis: (1) – ladder; (2,3) – pDNA; (4) – pLys+pDNA; (5) – pLys+pDNA+4xHep; (6) – pLys+pDNA+8xHep.

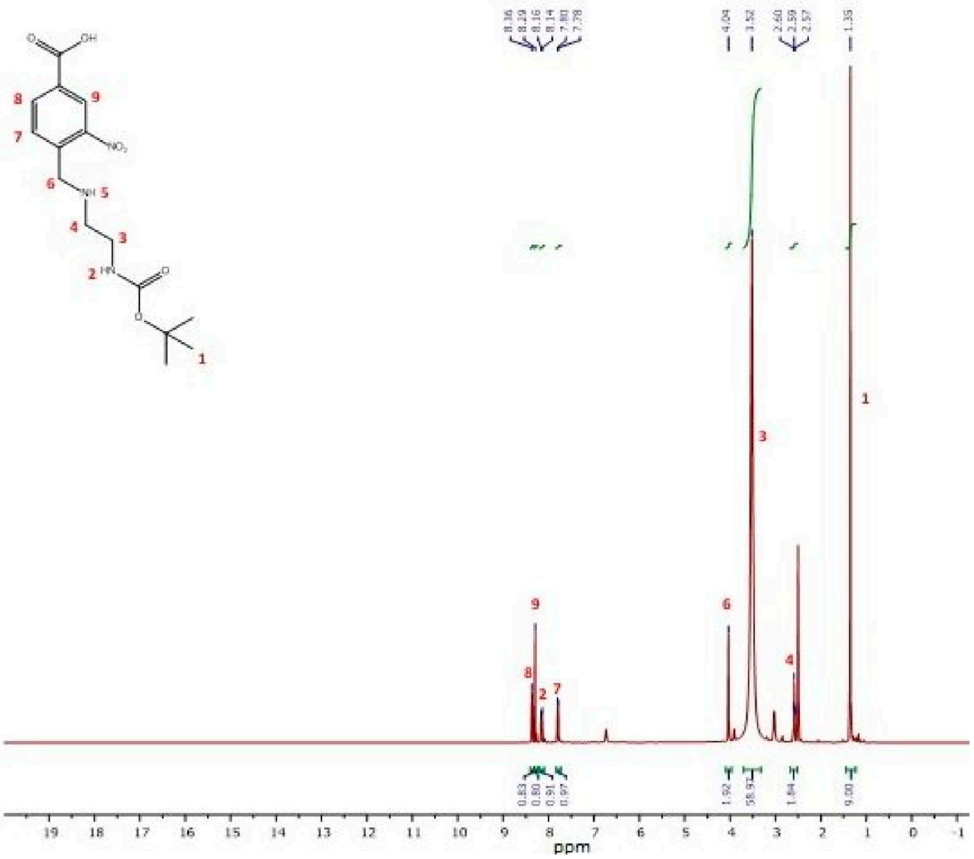

Figure S7. The ^{1}H NMR spectrum of 4 -(((2-((tert-butoxycarbonyl)amino) ethyl)amino)methyl)-3-nitrobenzoic acid.

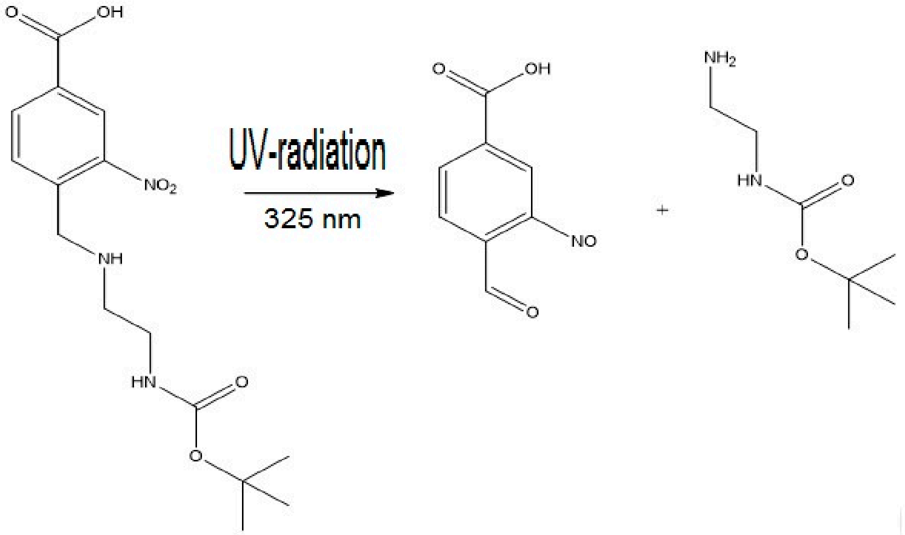

Figure S8. Linker (4 -(((2-((tert-butoxycarbonyl)amino)ethyl)amino)methyl)-3-nitrobenzoic acid decomposition under UV radiation at $\lambda = 325$ nm.

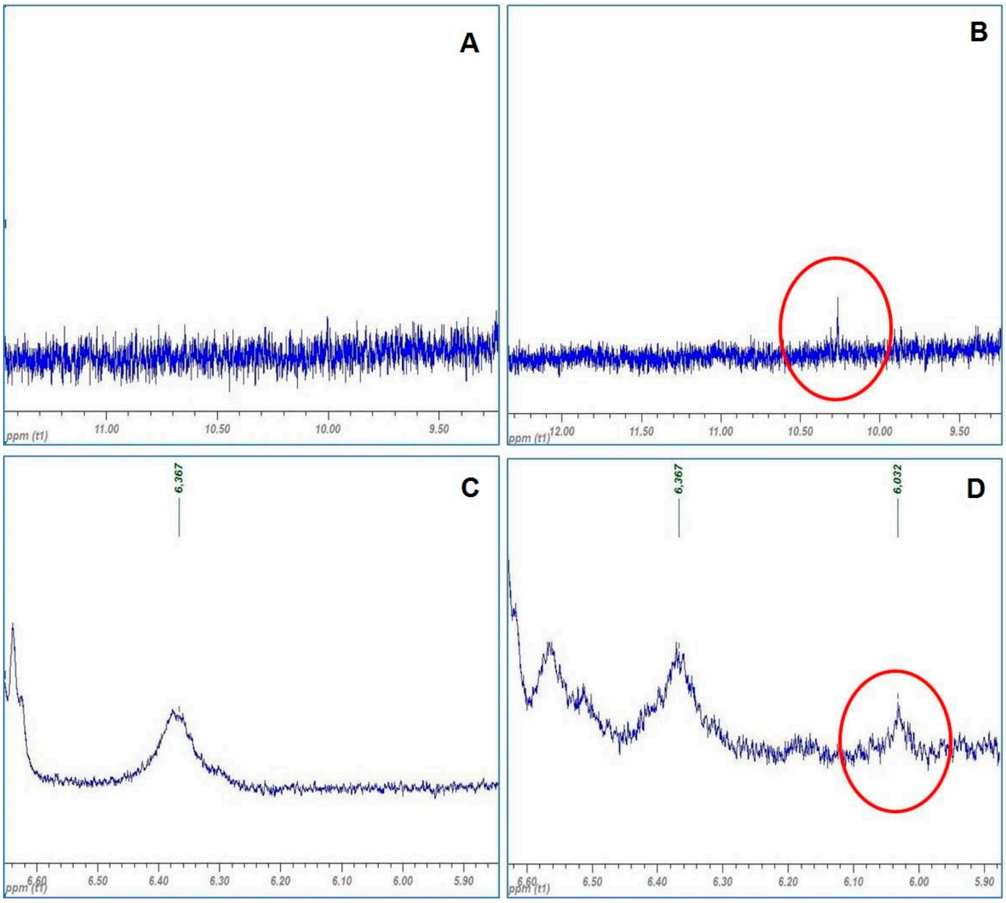

Figure S9. Differences in the ^1H NMR spectra of the initial linker (A, C) and its photodestruction product (B, D) when irradiated with a laser with a wavelength of 325 nm with a power of 3 W/cm^2 for 15 minutes. The signal of aldehyde (B) and hemiacetal (D) groups were detected. Spectra were obtained in DMSO-d6.

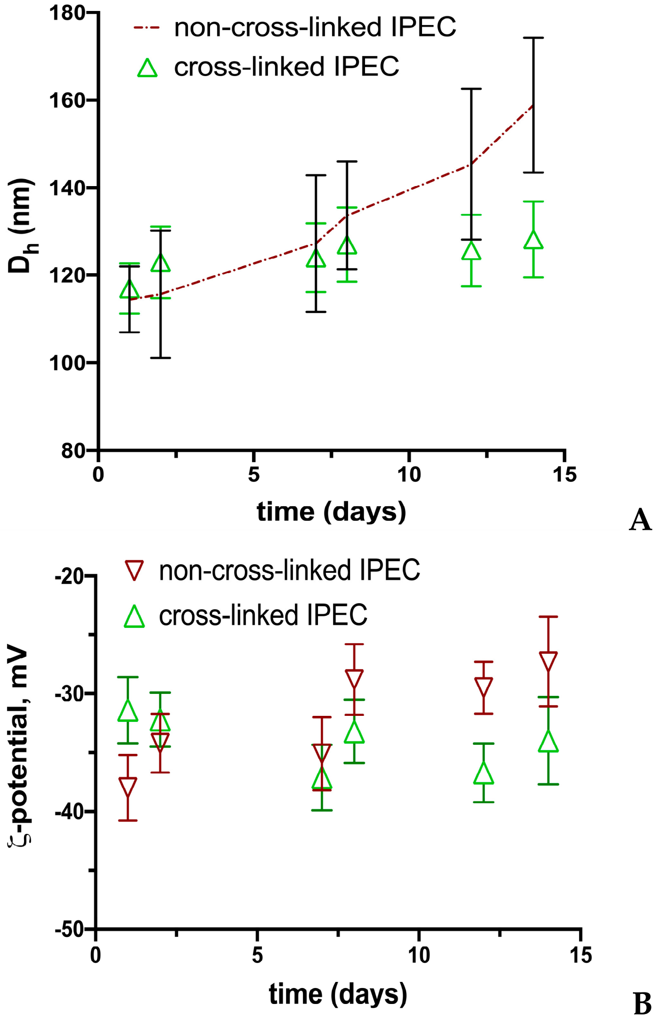

Figure S10. Stability of IPECS: particles hydrodynamic diameter (A) and ζ-potential (B) change with time. Conditions: 0.1 M PBS buffer solution, pH 7.4; particles concentration 0.1 mg/mL, 37 °C.

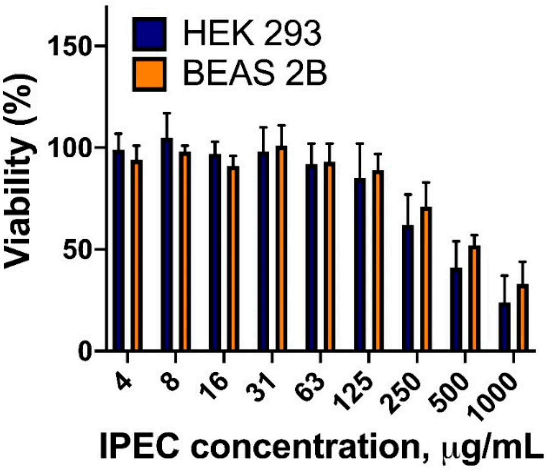

Figure S11. CTB test: viability of HEK 293 and BEAS 2B cells incubated with different concentrations of photo-sensitive linker (see **Figure 5**, compound **3**).

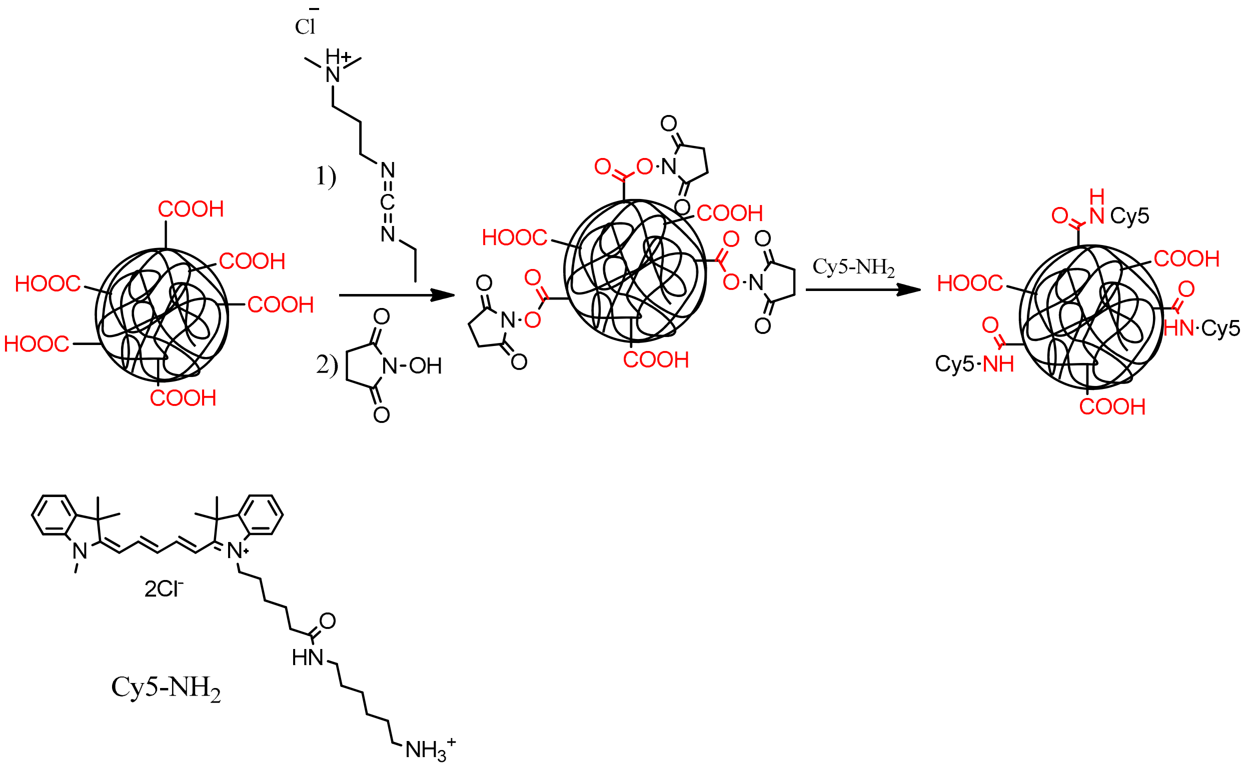

Figure S12. Modification of IPECs by amino-Cy5 probe.

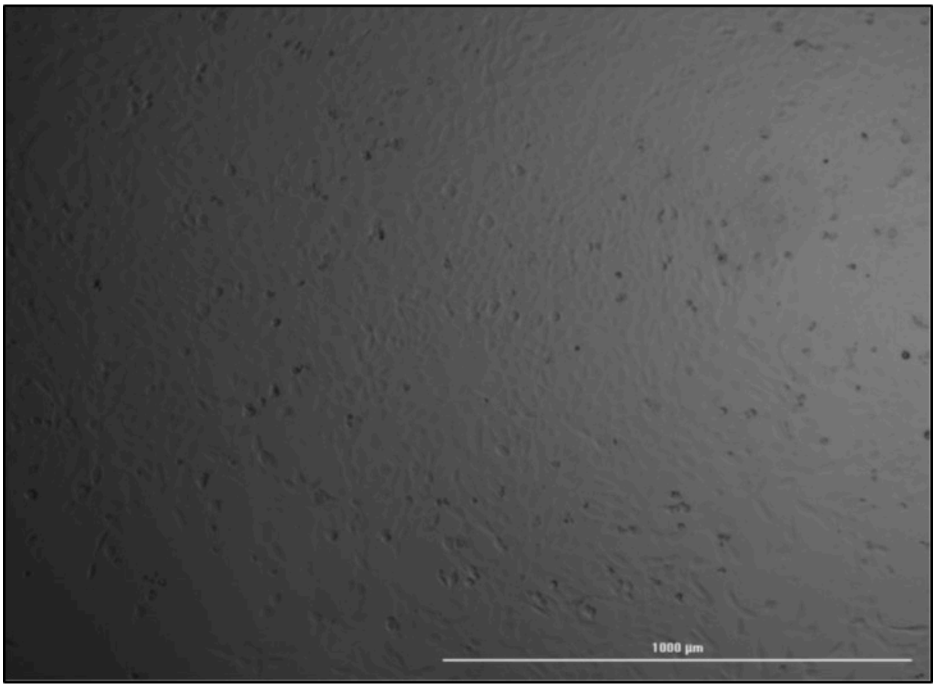

Figure S13. Optical microscopy: mouse fibroblast cells (NIH-3T3) morphology after exposure to 325 nm laser.
